# Full Genome Sequence-Based Comparative Study of Wild-Type and Vaccine Strains of Infectious Laryngotracheitis Virus from Italy

**DOI:** 10.1371/journal.pone.0149529

**Published:** 2016-02-18

**Authors:** Alessandra Piccirillo, Enrico Lavezzo, Giulia Niero, Ana Moreno, Paola Massi, Elisa Franchin, Stefano Toppo, Cristiano Salata, Giorgio Palù

**Affiliations:** 1 Department of Comparative Biomedicine and Food Science (BCA), University of Padua, Legnaro (Padua), Italy; 2 Department of Molecular Medicine, University of Padua (DMM), Padua, Italy; 3 Department of Virology, Istituto Zooprofilattico Sperimentale della Lombardia e dell’Emilia Romagna (IZSLER), Brescia, Italy; 4 Department of Diagnostics, Istituto Zooprofilattico Sperimentale della Lombardia e dell’Emilia Romagna (IZSLER), Forlì, Italy; Mathematical Institute, HUNGARY

## Abstract

Infectious laryngotracheitis (ILT) is an acute and highly contagious respiratory disease of chickens caused by an alphaherpesvirus, infectious laryngotracheitis virus (ILTV). Recently, full genome sequences of wild-type and vaccine strains have been determined worldwide, but none was from Europe. The aim of this study was to determine and analyse the complete genome sequences of five ILTV strains. Sequences were also compared to reveal the similarity of strains across time and to discriminate between wild-type and vaccine strains. Genomes of three ILTV field isolates from outbreaks occurred in Italy in 1980, 2007 and 2011, and two commercial chicken embryo origin (CEO) vaccines were sequenced using the 454 Life Sciences technology. The comparison with the Serva genome showed that 35 open reading frames (ORFs) differed across the five genomes. Overall, 54 single nucleotide polymorphisms (SNPs) and 27 amino acid differences in 19 ORFs and two insertions in the UL52 and ORFC genes were identified. Similarity among the field strains and between the field and the vaccine strains ranged from 99.96% to 99.99%. Phylogenetic analysis revealed a close relationship among them, as well. This study generated data on genomic variation among Italian ILTV strains revealing that, even though the genetic variability of the genome is well conserved across time and between wild-type and vaccine strains, some mutations may help in differentiating among them and may be involved in ILTV virulence/attenuation. The results of this study can contribute to the understanding of the molecular bases of ILTV pathogenicity and provide genetic markers to differentiate between wild-type and vaccine strains.

## Introduction

Infectious laryngotracheitis (ILT) is an acute and highly contagious respiratory disease of chickens caused by the *Gallid herpesvirus* 1 (GaHV-1), also called infectious laryngotracheitis virus (ILTV) [1]. ILTV is classified as a member of the genus *Iltovirus*, subfamily *Alphaherpesvirinae*, family *Herpesviridae* [2]. The virus has a linear dsDNA genome of about 150 kb composed of a unique long (UL), a unique short (US) region, and inverted internal (IR) and terminal (TR) repeats. The genome encodes 80 predicted viral protein open reading frames (ORFs): 65 ORFs are located within the UL region and nine within the US region, while the inverted repeats contain only three genes (ICP4, US10, and sORF4/3) [3].

Since its early appearance, several manifestations of ILT have been described associated with mortality and/or decreased egg production and causing significant economic losses to the poultry industry [1]. In commercial chicken flocks the disease is primarily controlled by vaccination and several types of vaccines have been produced, including killed, live attenuated and the recent recombinant vaccines [4]. Live attenuated vaccines are produced by sequential passages in tissue cultures (tissue culture origin, TCO) or embryonated eggs (chicken embryo origin, CEO) [1]. However, these vaccines may retain residual virulence and revert to virulence after bird-to-bird passage resulting in disease in unprotected flocks [5, 6].

In the last decade, several molecular studies [7–18] have provided evidence that strains identical or closely related to CEO vaccines have been involved in ILT outbreaks worldwide. These observations have been recently strengthened by full genome sequence-based analyses, which demonstrated that some reverted live attenuated vaccines were the main source of ILT outbreaks [4, 6]. A total of 22 full DNA sequences of wild-type and vaccine strains from Australia [3, 15, 19, 20], USA [16, 21, 22], and China [23] have been recently determined and are currently available at the NCBI GenBank nucleotide database. Except for the European Serva vaccine strain sequenced by an Australian research group [3], no full genome sequences of European ILTV strains have been determined to date.

In Italy, epidemics of ILT have followed a cyclical pattern. One severe epidemic caused by virulent ILTV strains occurred during the ‘80s [24]. Afterward, vaccination was introduced to control the disease, and ILTV disappeared to re-emerge in 2007 when a second epidemic of mild respiratory disease started to affect broiler flocks [25]. Currently, four ILT CEO vaccines are licensed in Italy and only long-living birds (*i*.*e*. breeders and layers) are routinely vaccinated. In a previous study [25], PCR-RFLP and sequencing analysis of six genes (*i*.*e*. gE, gG, TK, ICP4, ICP18.5, and ORFB-TK) suggested that some ILTV isolates involved in recent ILT outbreaks in Italy might have been originated from CEO vaccines.

In this study, the full genome sequences of five Italian ILTV strains were determined using the high throughput 454 Life Sciences sequencing technology. One field isolate from a historical case of ILT (1980), two field isolates from recent cases of ILT (2007 and 2011) and two commercially available CEO vaccine viruses (Nobilis Laringovac^®^, MSD Animal Health and Poulvac ILT^®^, Zoetis) were sequenced for analysing and comparing the genomes. The ultimate goal of this study was to identify genetic variation among wild-type isolates from different years and two distinct Italian epidemics of ILT, as well as between wild-type and vaccine strains.

## Materials and Methods

### Virus

Five ILTV strains were sequenced: one field isolate from a historical case of ILT (1980 ILTV field isolate, 4787/80), two field isolates from recent cases of ILT (2007 ILTV field isolate, 193435/07, and 2011 ILTV field isolate, 757/11), and two commercially available ILTV CEO vaccine viruses. The wild-type strains were isolated from unvaccinated chicken flocks affected by ILT in 1980, 2007 and 2011. The 1980 and 2007 ILTV strains were isolated from tracheal tissue propagated onto the chorioallantoic membrane (CAM) of SPF embryonated chicken eggs, whereas the 2011 strain was obtained from a tracheal swab. The two ILTV CEO vaccines were those commercialized in Italy as Nobilis Laringovac^®^ (MSD Animal Health S.r.l., Segrate [MI], Italy) and Poulvac ILT^®^ (Zoetis Italia S.r.l., Roma, Italy), attenuated by sequential passages in chicken embryos from the Serva and the Salsbury 146 strains, respectively.

### Viral DNA Extraction

Total viral genomic DNA was extracted directly from the suspension of the inoculated CAMs, the suspension of the tracheal swab and the reconstituted lyophilized vaccines using the High Pure Viral Nucleic Acid Kit (Roche Diagnostics S.p.A, Roche Applied Science, Monza [MI], Italy), according to the manufacturer’s instructions.

### High Throughput Sequencing

Viral genomic DNAs were preliminarily amplified by a conventional PCR in order to enrich the DNA by using the high fidelity Takara LA Taq^™^ DNA Polymerase (Lonza, Basel, Switzerland), allowing the generation of long and accurate PCR products. A set of about 10 kb overlapping amplicons was obtained with the primers summarized in S1 Table. Afterward, a shotgun sequencing approach was performed by using the GS Titanium Rapid Library Preparation Kit (Roche). Briefly, amplicons from each viral strain were first pooled equimolarly, and then fragmented by nebulization (at 3 psi for 1 minute), purified, and ligated to adapter-tags with barcodes. DNA fragments were clonally amplified and multiplex sequencing was performed by using the Roche 454 Life Sciences Genome Sequencer FLX platform (Roche 454 Life Sciences, Branford, CT, USA), following the manufacturer’s instructions.

### Sequencing Data Analysis

Images were processed using the runAnalysisPipe command provided with the DataProcessing package (Roche). The resulting sequences were demultiplexed using the SFFtools suite (Roche) and mapped to the ILTV reference sequence (Serva strain, GenBank accession no. HQ630064), using the gsMapper tool provided with the instrument. The same tool was used to detect variants between the ILTV strains sequenced in the study and the reference sequence.

### Whole Genome Finishing (Start/End)

The 5’ end of the ILTV genomes was amplified and sequenced with the Sanger method using a pair of primers (Forward: AATTTCCACCGCGAAATGGC; Reverse: AACCGGGGTTTAGAGCTGTG) designed to obtain a product of 1,292 bp. The 5’ and 3’ ends of the genomes were also determined following the protocol described by Kong et al. [23], consisting of a single oligonucleotide nested PCR (SON-PCR). Briefly, a single primer (GCGAGGTAGGGAGTGTGGCTGCTG) was used for the first step of the SON-PCR, whereas the forward primer (GGTCGGACATGAAACCACAAGG) mapping at the 3’ end and the reverse primer (TGGGTGCTTGCCTGCATATACC) mapping at the 5’ end of the genome were used for the nested PCR and sequencing.

The five Italian ILTV complete genomes were deposited in the NCBI GenBank database under the accession numbers: KP677881 (4787/80 field isolate), KP677882 (193435/07 field isolate), KP677883 (757/11 field isolate), KP677884 (MSD CEO vaccine) and KP677885 (Zoetis CEO vaccine).

### IR/TR Variant Characterization

Since the ILTV internal repeat (IR) and terminal repeat (TR) are almost identical, it was not possible to discriminate the reads from one region to those from the other one. To identify possible differences between the two regions, a modified version of the Serva genome (composed only by UL, IR, and US) was used as reference for mapping. All reads of both IR and TR were assembled and searched for variants with a consensus below 100% of the mapped reads, which could indicate mutations present only in one of the repeat regions, and further analysed with conventional PCR and Sanger sequencing. In particular, two couples of primers able to amplify selectively the IR region, were designed and subsequently targeted to the selected variants with nested PCRs (S2 Table). The detection of variants in the IR entailed their absence in the TR and vice versa.

### Alignment and Phylogenetic Analysis

A multiple alignment including the five ILTV genomes and all the ILTV complete genomes (except for the mosaic sequence of GaHV-1, accession number NC_006623) available in GenBank (Table 1) was built using ClustalW2 [26]. Similarly, the translated ORFs of all the genomes were analysed. Phylogenetic trees of selected genes were constructed with Mega 6 [27] using the Maximum Likelihood method based on the Tamura-Nei model [28].

**Table 1:** Detailed information of the ILTV complete genomes published in GenBank (accessed 2015 November 5) used in the alignment and phylogenetic analyses.

GenBank accession no.	Definition	Size (bp)	Virus strain		Vaccine name^a^	Country	Reference
JX458823	GaHV-1 strain WG	153,505	Virulent	WG	-	China	-
JX458824	GaHV-1 strain K317	153,639	Virulent	K317	-	China	-
JX458822	GaHV-1 strain LJS09	153,201	Virulent	LJS09	-	China	Zhao et al., 2013 [48]
JX646898	GaHV-1 strain V1-99	153,630	Virulent	V1-99	-	Australia	Lee et al., 2013
JX646899	GaHV-1 strain CSW-1	151,671	Virulent	CSW-1	-	Australia	Lee et al., 2013
JN580317	GaHV-1 strain CEO low passage	153,641	Vaccine	Hudson	Trachivax, Merck Animal Health	America	García et al., 2013b
JN580315	GaHV-1 strain TCO low passage	155,465	Vaccine	Unknown	LT-IVAX, Merck Animal Health	America	García et al., 2013b
JN580313	GaHV-1 strain CEO TRVX	153,647	Vaccine	Hudson	Trachivax, Merck Animal Health	America	García et al., 2013b
JN580316	GaHV-1 strain CEO high passage	153,647	Vaccine	Hudson	Trachivax, Merck Animal Health	America	García et al., 2013b
JN580314	GaHV-1 strain TCO high passage	150,335	Vaccine	Unknown	LT-IVAX, Merck Animal Health	America	García et al., 2013b
JN580312	GaHV-1 strain TCO IVAX	155,465	Vaccine	Unknown	LT-IVAX, Merck Animal Health	America	García et al., 2013b
JN804827	GaHV-1 strain CL9	152,635	Virulent	CL9	-	Australia	Lee et al., 2012
JN804826	GaHV-1 strain ACC78	152,632	Virulent	ACC78	-	Australia	Lee et al., 2012
JQ083493	GaHV-1 strain vaccine LT Blen	153,623	Vaccine	Cover	LT Blen, Merial	America	Chandra et al., 2012
JQ083494	GaHV-1 strain vaccine Laryngo Vac	153,624	Vaccine	Hudson	Laryngo-Vac, Zoetis	America	Chandra et al., 2012
JN542535	GaHV-1 strain 81658	150,335	Virulent	81658	-	America	Spatz et al., 2012
JN542533	GaHV-1 strain 1874C5	149,682	Virulent	1874C5	-	America	Spatz et al., 2012
JN542536	GaHV-1 strain 63140/C/08/BR	153,633	Virulent	63140/C/08/BR	-	America	Spatz et al., 2012
JN542534	GaHV-1 strain USDA reference	151,756	Virulent	USDA	-	America	Spatz et al., 2012
JN596963	GaHV-1 strain A20	152,978	Vaccine	A20	Poulvac Laryngo A20, Zoetis	Australia	Lee et al., 2011b
JN596962	GaHV-1 strain SA2	152,975	Vaccine	SA2	Poulvac Laryngo SA2, Zoetis	Australia	Lee et al., 2011b
HQ630064	GaHV-1 strain live attenuated Serva	152,630	Vaccine	Serva	Nobilis ILT, MSD	Australia	Lee et al., 2011a
NC_006623^b^	Gallid herpesvirus 1	148,687	-	-	-	-	Thureen & Keeler, 2006

^a^ Data obtained from Menendez et al., 2014;

^b^Sequence of ILTV assembled from 14 different published sequences.

This sequence was excluded from the multiple alignment and phylogenetic analyses.

## Results and Discussion

### Genome Organization

**Fig 1:**
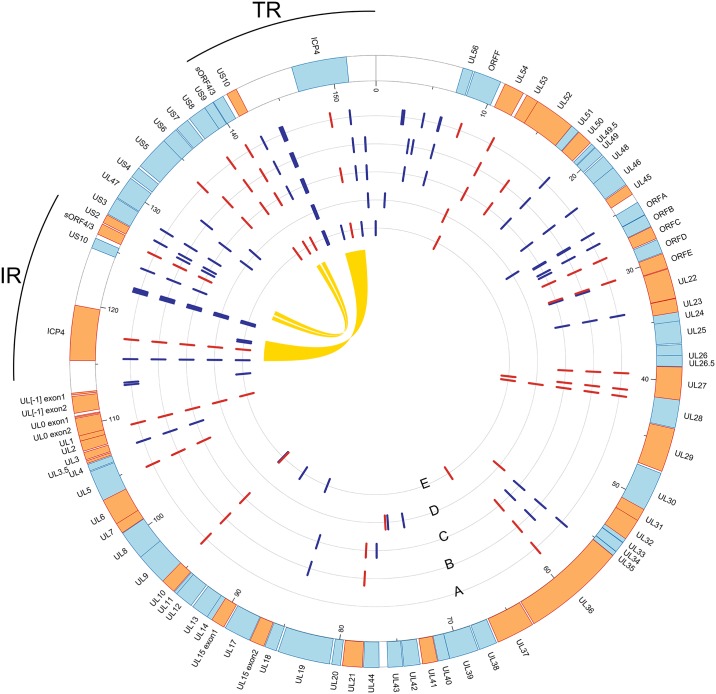
Representation of the nucleotide and amino acid differences detected in the five ILTV genomes. The outermost, thick circle is the NCBI ILTV reference sequence (Serva strain, GenBank accession no. HQ630064), where rectangles represent the coding sequences (CDS) and the colour code indicates the transcription strand: cyan refers to CDS in the plus strand, orange stands for minus strand. The inner circles represent the five ILTV genomes sequenced in this study (A = 4787/80, B = 193435/07, C = 757/11, D = MSD CEO vaccine, E = Zoetis CEO vaccine): on each circle, the blue bars indicate silent nucleotide substitutions, while red ones highlight those causing amino acid substitutions. The yellow ribbons in the inner part of the figure connect the coding sequences that are repeated in the ILTV genome. IR: internal repeat; TR: terminal repeat. The plot was obtained with Circos [44].

High throughput sequencing showed that the size of the five ILTV genomes ranged from 153,650 bp in the two vaccine strains to 153,653 bp in the 4787/80 field isolate and 153,662 bp in the other two field isolates. On average, the G + C content of the genomes was 47.97% and a total of 79 predicted ORFs was identified, according to the Serva reference sequence. A summary of the sequencing results, the full genome and genomic regions (UL, IR, US and TR) lengths and the G + C content of the five strains is shown in Table 2. In Fig 1, the alignment and the predicted ORFs arrangement of the five genomes with the Serva reference sequence are shown.

**Table 2:** Results of the pyrosequencing run and genomic assembly (NCBI ILTV Serva reference sequence is reported for comparison).

Strain	Number of reads	Average raw read length	Coverage	Total genome length (bp)	UL (bp)	IR (bp)	US (bp)	TR (bp)	G + C content
4787/80 field isolate	44,175	543.27^a^	156X	153,653	112,917 (1–112,917)	13,821 (112,918–126,738)	13,094 (126,739–139,832)	13,821 (139,833–153,653)	48.11%
193435/07 field isolate	15,657	372.42	38X	153,662	112,926 (1–112,926)	13,821 (112,927–126,747)	13,094 (126,748–139,841)	13,821 (139,842–153,662)	48.1%
757/11 field isolate	26,867	376.86	66X	153,662	112,926 (1–112,926)	13,821 (112,927–126,747)	13,094 (126,748–139,841)	13,821 (139,842–153,662)	48.1%
MSD CEO vaccine strain	22,433	377.86	55X	153,650	112,914 (1–112,914)	13,821 (112,915–126,735)	13,094 (126,736–139,829)	13,821 (139,830–153,650)	48.11%
Zoetis CEO vaccine strain	53,001	385.71	133X	153,650	112,914 (1–112,914)	13,821 (112,915–126,735)	13,094 (126,736–139,829)	13,821 (139,830–153,650)	48.11%
Serva vaccine strain	-	-	-	153,645	112,915	13,818	13,094	13,818	48%

UL = unique long; IR = internal repeat; US = unique short; TR = terminal repeat; G + C = guanine + cytosine content.

^a^Average read length is considerably higher for the strain 4787/80 because sequencing was conducted on a separate run, after the upgrade of the instrument to FLX Plus.

Significant differences in the ILTV full genome lengths (ranging from 150,335 to 155,465 bp) have been reported [3, 15, 16, 19–23], with the extreme values due to large insertions/deletions (INDELs), such as the deletion of about 3.3 kb at the 5’ end of the US TCO vaccines and related field strains [16, 21]. Instead, differences in the genome length of the five strains sequenced in this study did not involve large fragments of DNA. Accordingly with Spatz et al. [21], a possible error in the assembly of the Serva reference genome was detected: a fragment of 1,016 bp was missing at the 3’ end of the TR region leading to an increase in the size of the Serva genome from 152,630 to 153,645 bp. Additionally, the lack of this sequence fragment was identified in the genomes of the Aus CEO vaccines (SA2 and A20), the Aus virulent strains (ACC78 and CL9), and the US 1874C5 field strain.

Overall, the multiple amino acid sequence alignment showed that only 11 ORFs presented different lengths among the ILTV genomes. Compared to the Serva reference sequence, differences in the coding regions of the five strains consisted of an insertion of nine nucleotides (CCTCCTCTT, after nt position 1,892; Δ491GluGluGlu) in the UL52 gene of two field strains (193435/07 and 757/11) and an insertion of three nucleotides (ATC, after nt position 813; Δ66Asp) in the ORFC gene of the three field strains. The sequence alignment showed that the insertion of three Glu in the UL52 gene was shared by the two Italian field strains and the Aus V1-99 virulent strain. Notably, this insertion was located in a low complexity region rich of CCT or CTT codons, suggesting a possible replication slippage event. The insertion in ORFC was also present in the US 63140 isolate, a virulent strain closely related to CEO vaccines, in the Aus CEO vaccines (SA2 and A20) and virulent strains (CL9 and CSW-1), and in the Chi WG field strain. Both these mutations in UL52 and ORFC genes should be further investigated in order to clarify their potential impact on viral biology and pathogenesis.

### Comparative Analysis

Comparative analysis between the five ILTV strains and the Serva reference sequence showed that the nucleotide sequence identity ranged from 99.96% to 99.99%, with the lowest value detected in the 193435/07 and 757/11 field isolates. The DNA sequence identity among the wild-type strains and among the vaccine strains was 99.99% and 99.98%, respectively, whereas that between them ranged from 99.97% to 99.98% (Table 3). All nucleotide and predicted amino acid differences in the coding regions of the five strains compared to the Serva reference sequence are presented in Table 4. Besides the two insertions identified in the UL52 and ORFC genes, nucleotide differences were located in a total of 35 ORFs (including IR and TR variants). The remaining 44 ORFs were completely conserved across the genomes. These findings are consistent with previous studies reporting a pronounced genetic stability in ILTV [3, 16, 19–22], according to the low mutation rate in members of the *Alphaherpesvirinae* subfamily [29]. The multiple amino acid sequence alignment showed that six ORFs were completely conserved among the ILTV genomes. A 100% identity in ORFs of virulent, TCO and CEO vaccine strains from distant geographical areas (*i*.*e*. USA, Australia and China) has been documented previously [16, 19, 20–23]. The conservation of genes in ILTV strains divergent for geographical origin and virulence suggests that they may not be related to the virulence/attenuation of the virus.

**Table 3:** Pairwise comparison of the five ILTV strains with the NCBI ILTV reference sequence (Serva strain) (global identity percentage and number of identities over the genome length of the shortest of the two).

	MSD CEO	Zoetis CEO	193435/07	757/11	4787/80	Serva strain
**MSD CEO**	100%					
**Zoetis CEO**	99.98% (153,625/153,650)	100%				
**193435/07**	99.97% (153,605/153,650)	99.97% (153,610/153,650)	100%			
**757/11**	99.97% (153,603/153,650)	99.97% (153,610/153,650)	99.99% (153,660/153,662)	100%		
**4787/80**	99.97% (153,611/153,650)	99.98% (153,619/153,650)	99.99% (153,638/153,653)	99.99% (153,638/153,653)	100%	
**Serva strain**	99.98% (153,609/153,645)	99.97% (153,606/153,645)	99.96% (153,588/153,645)	99.96% (153,586/153,645)	99.97% (153,593/153,645)	100%

**Table 4:** Summary of nucleotide and amino acid differences in the coding regions of the five ILTV strains *vs* the genome sequence of the NCBI ILTV reference sequence (Serva strain).

Gene		CDS			Genomic nt position	Nt substitution (strand +)^a^	Field strains^b^			Vaccine strains^b^	
Name	Product	Nt position (strand +)	Nt position (coding strand)	AA position			4787/80	193435/07	757/11	MSD CEO	Zoetis CEO
**ORFF**	**Protein IF**	**437**	**437**	**146**	**8,453**	**T → C**	**V → A**	-	-	-	-
**UL54**	**Multifunctional expression regulator**	**199**	**1,437**	**479**	**10,844**	**T → C**	-	-	-	**I → M (60%)**	-
		**913**	**723**	**241**	**11,558**	**T → G**	-	-	-	-	**E → D**
		**1,235**	**401**	**134**	**11,880**	**C → T**	**S → N**	**S → N**	**S → N**	-	-
**UL52**	**Helicase-primase primase subunit**	**1,892**	**1,442**	**481**	**15,495**	**- → CCTCCTCTT**	-	**Q → QEEE**	**Q → QEEE**	-	-
UL50	Deoxyuridine triphosphatase	799	453	151	18,490	A **→** G	T **→** T	T **→** T	T **→** T	-	-
UL46	Tegument protein VP11/12	849	849	283	22,496	A **→** G	T **→** T	T **→** T	T **→** T	-	-
ORFA	Protein IA	360	360	120	25,397	A **→** C	S **→** S	S **→** S	S **→** S (90.9%)	-	-
		465	465	155	25,502	C **→** T	-	V **→** V	V **→** V (94.6%)	-	-
ORFB	Protein IB	352	352	118	26,559	A **→** C	R **→** R	R **→** R	R **→** R	-	-
		744	744	248	26,951	T **→** C	-	-	A **→** A	-	-
**ORFC**	**Protein IC**	**813**	**197**	**66**	**28,097**	**- → ATC**	**D → DD (90.3%)**	**D → DD**	**D → DD**	-	-
**ORFE**	**Protein IE**	**836**	**398**	**133**	**30,430**	**C → G**	**G → A**	**G → A**	**G → A**	-	-
		1042	192	64	30,636	C **→** T	-	S **→** S	S **→** S	-	-
UL23	Thymidine kinase	553	540	180	33,885	G **→** A	D → D	D → D	D → D	-	-
**UL27**	**Envelope glycoprotein B**	**722**	**1931**	**644**	**39,573**	**A → G**	**I → T**	**I → T**	**I → T**	-	-
		**2306**	**347**	**116**	**41,157**	**A → G**	**V → A**	**V → A**	**V → A**	-	**V → A (95.1%)**
**UL28**	**DNA packaging terminase subunit 2**	**383**	**1913**	**638**	**41,857**	**A → G**	**V → A**	**V → A**	**V → A**	**V → A (66.7%)**	**V → A**
**UL36**	**Large tegument protein**	**413**	**7943**	**2648**	**55,613**	**C → T**	-	-	-	**R → H (33.3%)**	-
		679	7677	2559	55,879	T → C	T → T (98.3%)	T → T	T → T	-	-
		2386	5970	1990	57,586	C → T	-	G → G	G → G	-	-
		**4316**	**4040**	**1347**	**59,516**	**C → T**	**R → H**	**R → H**	**R → H**	-	-
		**7532**	**824**	**275**	**62,732**	**G → A**	-	-	-	-	**A → V (65.2%)**
UL41	Tegument host shutoff protein	832	369	123	72,697	A → G	-	-	-	R → R (10.3%)	-
**UL43**	**Envelope protein UL43**	369	369	123	75,044	T **→** C	-	-	-	F → F	-
		**761**	**761**	**254**	**75,436**	**C → T**	-	-	-	**T → I (36.8%)**	-
UL44	Envelope glycoprotein C	180	180	60	76,749	A → G	-	-	E → E (10.3%)	-	-
**UL21**	**Tegument protein UL21**	**211**	**1389**	**463**	**78,110**	**C → A**	-	**K → N**	**K → N**	-	-
UL19	Major capsid protein	3870	3870	1291	84,421	C → T	-	L → L	L → L	-	-
UL14	Tegument protein UL14	459	459	153	90,940	A → G	-	-	-	-	L → L
**UL10**	**Envelope glycoprotein M**	**1059**	**124**	**42**	**95,522**	**T → C**	**T → A**	**T → A**	**T → A**	-	**T → A (56%)**
UL9	DNA replication origin-binding helicase	78	78	26	95,671	T → C	-	-	-	-	P → P
**UL5**	**Helicase-primase helicase subunit**	**1027**	**1027**	**343**	**104,708**	**A → G**	**K → E**	**K → E**	**K → E**	-	-
**UL1**	**Envelope glycoprotein L**	**287**	**161**	**54**	**109,069**	**T → G**	**Q → P**	**Q → P**	**Q → P**	**Q → P**	**Q → P**
**ICP4**	**Transcriptional regulator ICP4**	169	4281	1427	115,469	C → T	G → G	G → G	G → G	G → G	G → G
		**2108**	**2342**	**781**	**117,408**	**T → C**	**H → R**	**H → R**	**H → R**	**H → R**	**H → R**
		3460	990	330	118,760	T → C	-	-	-	-	S → S
		3778	672	224	119,078	T → C	-	-	-	-	E → E
**sORF4/3**	**Protein sORF4/3**	**444**	**442**	**148**	**126,167**	**C → T**	**A → T**	**A → T**	**A → T**	-	-
		**452**	**434**	**145**	**126,175**	**C → A**	-	**R → L**	**R → L**	-	-
US2	Virion protein US2	289	402	134	127,028	G → A	-	F → F	F → F	-	-
US3	Serine/threonine protein kinase US3	1200	1200	400	128,717	A → G	G → G	G → G	G → G	-	-
UL47	Tegument protein VP13/14	1812	1812	604	130,845	A → G	-	-	-	S → S (47.6%)	-
US4	Envelope glycoprotein G	33	33	11	131,101	C → T	-	-	A → A (16%)	-	-
		765	765	255	131,833	G → A	-	E → E	-	-	-
**US5**	**Envelope glycoprotein J**	777	777	259	132,933	T → C	-	-	-	I → I (31.6%)	-
		**2345**	**2345**	**782**	**134,501**	**A → G**	**D → G (10%)**	-	-	-	-
**US6**	**Envelope glycoprotein D**	**401**	**401**	**134**	**135,322**	**C → T**	-	**S → L**	**S → L**	-	-
**US8**	**Envelope glycoprotein E**	**629**	**629**	**210**	**138,244**	**A → G**	**K → R**	**K → R**	**K → R**	-	**K → R**
**sORF4/3**	**Protein sORF4/3**	**307**	**307**	**103**	**140,259**	**C->T**	-	-		-	**P → S (10.7%)**
		**434**	**434**	**145**	**140,386**	**G->T**	-	**R → L**	**R → L**	-	-
		**442**	**442**	**148**	**140,394**	**G->A**	**A → T**	**A → T**	**A → T**	-	-
**US10**	**Virion protein US10**	**489**	**349**	**117**	**141,692**	**C->T**	-	-	-	-	**A → T (35.3%)**
**ICP4**	**Transcriptional regulator ICP4**	672	672	224	147,483	A->G	-	-	-	-	E → E
		**2342**	**2342**	**781**	**149,153**	**A->G**	**H → R**	-	**H → R**	-	**H → R**
		4281	4281	1427	151,092	G->A	G → G	G → G	G → G	-	G → G

CDS = coding DNA sequence; Nt = nucleotide; AA = amino acid.

^a^Nucleotide in the reference sequence (Serva strain);

^b^Predicted AA; Percentages indicate the amount of reads bearing the variant (not reported when 100%).

In bold non-synonymous nt substitutions.

Comparing the three field isolates with the Serva reference sequence, a total of 38 SNPs and two insertions were identified (Table 4). Eighteen SNPs were synonymous and 20 non-synonymous. Thirteen non-synonymous SNPs were found in all the three wild-type strains. Non-synonymous SNPs in ORFF (T437C; Val146Ala) and US5 (A777G; Asp259Gly) were detected only in the 4787/80 isolate; SNPs in UL21 (C1389A; Lys463Asp), sORF4/3-IR/TR (C434A; Arg145Leu and G434T, Arg145Leu, respectively), and US6 (C401T; Ser134Leu) only in the 193435/07 and 757/11 isolates; and SNP in ICP4-TR (A2342G; His781Arg) only in the 4787/80 and 757/11 isolates. The comparison between the two vaccine strains and the Serva reference sequence revealed a total of 25 SNPs (Table 4). Eleven SNPs were synonymous and 14 non-synonymous. Three non-synonymous SNPs were detected in both the vaccine strains, as well as in the three wild-type isolates. Three SNPs were unique to the MSD CEO vaccine strain, while eight were detected only in the Zoetis CEO vaccine. SNPs in UL27, UL10, and US8 were also present in the three wild-type strains, whereas the nucleotide substitution in ICP4-TR was present only in two (4787/80 and 757/11) of the three field isolates. Notably, some non-synonymous SNPs were present only in a portion of reads supporting the hypothesis of the existence of viral subpopulations [16, 22]. Nucleotide and amino acid differences were also identified between the two repeat regions (IR and TR) of the five strains (Table 4). To our knowledge, no other studies have reported any difference between the IR and TR of ILTV genome.

In Table 5, amino acid differences in the coding regions of the five strains compared to the complete ILTV genomes available in GenBank are summarized. Among 12 ILTV genes encoding for surface glycoproteins [1], six (*i*.*e*. UL27, UL10, UL1, US5, US6, and US8 encoding for gB, gM, gL, gJ, gD, and gE, respectively) revealed at least one amino acid change, and many of these mutations were also present in other ILTV strains. Spatz et al. [21] suggested that mutations in the surface glycoproteins gB, gM, gE and gL might have been caused by the different geographical origin of the European Serva strain and the US virulent strains. Interestingly, we found these mutations either in European ILTV field and CEO vaccine strains, including the historical 4787/80 isolate. Therefore, mutations in the surface glycoproteins may not only have been caused by the geographical pressure, but they may also be related to virulence/attenuation of ILTV, given the important role these proteins play in host range and pathogenicity [30]. Besides the mutation Gln54Pro in the UL1 gene present in all the ILTV strains (except for the Serva strain and the Chi WG and K317 strains), most of the mutations (*i*.*e*. UL27 Val116Ala, UL10 Thr42Ala, and US8 Lys210Arg) were identified either in virulent and attenuated CEO and TCO vaccine strains. Therefore, they do not seem to be useful for discriminating between wild-type and vaccine ILTV strains. On the other hand, the mutation Ile644Thr in the UL27 gene was shared by all the virulent strains (except for the Chi LJS09 and K317) and lacking in all the vaccine strains (except for the Aus SA2 and A20), as previously documented by García et al. [16]. This mutation indeed appears to be a good marker for discriminating between field and vaccine strains. It should be mentioned that glycoprotein B (encoded by the UL27 gene) plays a fundamental role in herpesvirus attachment to target cells and cell entry [31]; then it could affect viral tropism and infectivity. The mutations found in the US5 and US6 genes were exclusive of the strains sequenced in this study and not reported previously. Notably, the mutation in the US6 gene was shared only by the two recent field isolates (193435/07 and 757/11). This gene encodes for the surface glycoprotein D that functions as receptor for herpesvirus and ILTV binding and entry to susceptible cells [32, 33]. Additionally, this protein acts synergistically with the glycoprotein J (encoded by the US5 gene), which plays a key role in herpesvirus infection by blocking the apoptotic cascade [34], affecting the virus ability to infect productively target cells.

**Table 5:** Summary of the amino acid differences in coding regions of the five ILTV strains *vs* other ILTV complete genomes published in GenBank (accessed 2015 November 5).

Gene	AA position	4787/80 virulent	193435/07 virulent	757/11 virulent	MSD CEO vaccine	Zoetis CEO vaccine	Serva CEO vaccine	SA2 CEO vaccine	A20 CEO/TCO vaccine	USDA virulent	63140 virulent	1874C5 virulent	81658 virulent	ACC78 virulent	CL9 virulent	LT-Blen CEO vaccine	Laryngo-Vac CEO vaccine	Trachivax CEO vaccine	LT-IVAX TCO vaccine	V1-99 virulent	CSW-1 virulent	LJS09 virulent	WG virulent	K317 virulent
**ORFF**	146	A	V	V	V	V	V	V	V	V	V	V	V	V	V	V	V	V	V	V	V	V	V	V
**UL54**	479	I	I	I	M	I	I	I	I	I	I	I	I	I	M	M	I	M	I	I	I	M	M	I
	241	E	E	E	E	D	E	E	E	E	E	E	E	E	E	E	D	E	E	E	E	E	E	E
	134	N	N	N	S	S	S	S	S	S	S	S	S	S	S	S	S	S	S	S	S	S	S	S
**UL52**	491	Q	QEEE	QEEE	Q	Q	Q	Q	Q	Q	Q	Q	Q	Q	Q	Q	Q	Q	Q	QEEE	Q	Q	Q	Q
**ORFC**	66	DD	DD	DD	D	D	D	DD	DD	D	DD	D	D	D	DD	D	D	D	D	D	DD	D	DD	D
**ORFE**	133	A	A	A	G	G	G	G	G	G	A	G	G	G	G	G	G	G	G	G	G	G	G	G
**UL27**	644	T	T	T	I	I	I	T	T	T	T	T	I	T	T	I	I	I	I	T	T	I	T	I
	116	A	A	A	V	A	V	A	A	A	A	A	A	A	A	A	A	A	A	A	A	V	A	A
**UL28**	638	A	A	A	A	A	V	A	A	A	A	A	A	A	A	A	A	A	A	A	V	V	A	A
**UL36**	2648	R	R	R	H (33.3%)	R	R	R	R	R	R	R	R	R	R	R	R	R	R	R	R	H	R	R
	1347	H	H	H	R	R	R	H	H	R	H	H	R	R	H	R	R	R	R	H	H	R	H	R
	275	A	A	A	A	V	A	A	A	A	A	A	A	A	A	A	V	A	A	A	A	A	A	A
**UL43**	254	T	T	T	I (36.8%)	T	T	T	T	T	T	T	T	T	T	T	T	T	T	T	T	I	T	T
**UL21**	463	K	N	N	K	K	K	K	K	K	K	K	K	K	K	K	K	K	K	K	K	K	K	K
**UL10**	42	A	A	A	T	A	T	A	A	A	A	A	A	T	A	T	T	T	A	A	A	T	T	T
**UL5**	343	E	E	E	K	K	K	E	E	E	E	E	E	K	E	K	K	K	E	E	E	K	E	K
**UL1**	54	P	P	P	P	P	Q	P	P	P	P	P	P	P	P	P	P	P	P	P	P	P	Q	Q
**ICP4**	781	R	R	R	R	R	H	R	R	R	R	R	R	H	H	R	R	R	R	R	R	H	R	R
**sORF4/3**	148	T	T	T	A	A	A	A	A	A	A	A	A	A	A	A	A	A	A	A	A	A	A	A
	145	R	L	L	R	R	R	R	R	R	R	R	R	R	R	R	R	R	R	R	R	R	R	R
**US5**	782	G (10%)	D	D	D	D	D	D	D	D	D	D	D	D	D	D	D	D	D	D	D	D	D	D
**US6**	134	S	L	L	S	S	S	S	S	S	S	S	S	S	S	S	S	S	S	S	S	S	S	S
**US8**	210	R	R	R	K	R	K	R	R	R	R	R	R	K	K	K	R	K	R	R	R	K	R	K
**sORF4/3**	103	P	P	P	P	S (10.7%)	P	P	P	P	P	P	P	P	P	P	P	P	P	P	P	P	P	P
	145	R	L	L	R	R	R	R	R	R	R	R	R	R	R	R	R	R	R	R	R	R	R	R
	148	T	T	T	A	A	A	A	A	A	A	A	A	A	A	A	A	A	A	A	A	A	A	A
**US10**	117	A	A	A	A	T	A	A	A	A	A	A	A	A	A	A	T	A	A	A	A	A	A	A
**ICP4**	781	R	H	R	H	R	H	R	R	R	R	R	R	H	H	R	R	R	R	R	R	H	R	R

AA = amino acid; Percentages indicate the amount of reads bearing the variant (not reported when 100%). Strains and GenBank accession numbers: Serva (HQ630064), SA2 (JN596962), A20 (JN596963), USDA (JN542534), 63140 (JN542536), 1874C5 (JN542533), 81658 (JN542535), ACC78 (JN804826), CL9 (JN804827), LT-Blen (JQ083494), Laryngo-Vac (JQ083493), Trachivax (JN580312), LT-IVAX (JN580313), V1-99 (JX646898), CSW-1 (646899), LJS09 (JX458822), WG (JX458823), and K317 (JX458824).

Non-synonymous SNPs were found among genes encoding for viral enzymes, which play a fundamental role during the replication cycle of herpesviruses by mediating the synthesis of viral DNA [35]. Mutations were found in the UL28 gene encoding for the DNA packaging terminase subunit 2, and the UL5 gene encoding for the helicase-primase helicase subunit. The mutation in the UL5 gene was found mainly in field strains, appearing to be discriminating between wild-type and vaccine strains, whereas that in the UL28 gene was found in most of the ILTV strains including field, CEO and TCO vaccine strains. Additionally, an insertion in the UL52 gene was uniquely identified in the two recent field isolates (193435/07 and 757/11) and in the Aus V1-99 virulent strain. Together with the UL5 and UL8 genes, the UL52 gene encodes for a component of the helicase-primase complex, which is essential for DNA replication fidelity of herpesviruses and thus for virus propagation [36]. Therefore, the biological significance of such mutation requires further investigation in order to determine if it may be involved in the genome stability and in the modulation of the virulence of the recent Italian ILTV field strains.

Among genes encoding for ILTV transcriptional regulatory proteins, ICP4 and UL54 showed amino acid differences. In the ICP4 gene, only a single amino acid change was identified in the five genomes, and it was shared by most of the strains including field, CEO and TCO vaccine strains. Among the three amino acid changes identified in the UL54 gene, the Ile479Met was present only in the MSD CEO vaccine and the Glu241Asp only in the Zoetis CEO vaccine. Similarly, Chandra et al. [22] found the same mutations in the LT-Blen and Laryngo-Vac CEO vaccines and García et al. [16] in the Trachivax CEO vaccine. Interestingly, the third mutation (Ser134Asn) was exclusive of the three field isolates sequenced in this study. The UL54 gene encodes for the infected cell protein 27, a multifunctional regulatory protein functioning in all stages of mRNA biogenesis from transcription, RNA processing and export through translation [37]. Therefore, the Ser134Asn mutation could have an impact on the efficiency of the viral replication cycle. In addition, it seems to be useful for discriminating between the Italian field and vaccine strains.

Amino acid changes were also found in genes encoding for structural proteins, such as the viral tegument proteins UL36 (large tegument protein) and UL21 (tegument protein UL21), and encoding for the viral capsid protein UL43 (envelope protein UL43). Among all the mutations detected in the UL36 gene, only the Arg1347His has been already described in the US 63140 field strain [16]. The UL36 gene encodes for the largest tegument protein of ILTV, which has been demonstrated essential for virion assembly in herpesviruses, particularly for the formation of structurally normal connections to the capsid in the initial stages of tegument addition [38]. In this study, the mutation was identified in all the three ILTV field strains and in other wild-type ILTV strains, appearing discriminating between field and vaccine strains. As for the UL54 gene, the Ala275Val mutation was identified only in the Zoetis CEO vaccine sequenced in this study and in the US Laryngo-Vac CEO vaccine sequenced by Chandra et al. [22]. The mutation in the UL21 gene was exclusive of the two recent field strains (193435/07 and 757/11) and not reported previously. The UL21 gene encodes for a conserved protein of the alphaherpesvirus tegument, essential for virus propagation and expressed during the early and late stages of the replication cycle [39]. Tegument addition in herpesvirus morphogenesis is a complex event involving several proteins. In addition, proteins of the tegument can have regulatory or enzymatic activities. Mutations on these proteins can affect the efficiency of viral assembly and the ability to produce an infective viral progeny [38, 39]. The mutation in the UL43 gene was found exclusively in the MSD CEO vaccine and the Chi LJS09 field strain.

A number of non-synonymous SNPs and an insertion was found in the ORFF, ORFC, ORFE, sORF4/3 and US10 genes, whose function is still unknown [1]. The ORFC and ORFE genes are known to be unique genes of the members of the *Iltovirus* genus [30] and previous studies carried out by Veits et al. [40] demonstrated that they are expressed during infection, but not essential for ILTV replication. As for the insertion in the ORFC gene of the three field strains, in the ORFE gene a mutation exclusive of these strains and the US 63140 isolate was found. Our finding is in agreement with Spatz et al. [21] and García et al. [16], who suggested that it may discriminate between the US 63140 CEO-related field strain and the US CEO vaccine strains and be involved in ILTV attenuation/virulence. In the ORFF gene, a single mutation was found only in the 4787/80 strain and not in the other strains. Similarly to other genes (*i*.*e*. UL54 and UL36), the mutation in the US10 gene was found only in the Zoetis CEO vaccine sequenced in this study and in the US Laryngo-Vac CEO vaccine. The two mutations in the sORF4/3 gene were present only in the Italian field strains: the Arg145Leu in all the three field isolates, the Ala148Thr only in the two recent isolates (193435/07 and 757/11).

Although several mutations were identified only in field strains, allowing the possibility to discriminate between field and vaccine strains in epidemiological studies, it is needed to clarify their physiological function and their potential impact on ILTV biology to identify the genetic determinants of ILT pathogenesis. In particular, it is essential the characterization of the function of the unique genes of the *Iltovirus* genus members that could play a key role on the modulation of the virulence.

Interestingly, the two Italian CEO vaccines (MSD and Zoetis) were more closely related to the US CEO vaccines than to the European Serva strain. In particular, the MSD CEO vaccine was almost identical to the US LT-Blen and Trachivax CEO vaccines, and the Zoetis CEO vaccine to the US Laryngo-Vac CEO vaccine. This finding needs to be further investigated since the Italian MSD CEO vaccine is reported to contain the Serva strain and the Italian Zoetis CEO vaccine the Salsbury strain, whereas the US CEO vaccines are reported to contain the Hudson strain (LT-Blen, Merial Select and Trachivax, Merck Animal Health) and the Cover strain (Laryngo-Vac, Fort-Dodge Animal Health) [3, 16, 22].

Although a very high sequence identity among the five ILTV strains was detected, the most relevant finding of this study was the pronounced genetic similarity (99.99%) between the historical field strain and the two recent field isolates (193435/07 and 757/11). The 4787/80 strain was isolated during a severe ILT outbreak occurred in the ‘80s when the vaccination against ILT had not yet been introduced in Italy [24]. The other two strains were instead isolated from outbreaks of ILT occurred recently (2007 and 2011, respectively) in unvaccinated broiler flocks, showing mild clinical signs of the disease [25]. In a previous study [25], the Authors postulated that the recent Italian ILT outbreaks were caused by strains closely related to CEO vaccines based on the results of a multi-loci PCR-RFLP and sequencing of a number of field isolates and CEO vaccines. Interestingly, analysis of partial sequences discriminated between them. Most of the studies aimed at differentiating ILTV wild-type and vaccine strains have been based on single- or multi-locus PCR-RFLP [10, 12, 13, 17, 41, 42] and sequencing [11] or both [7–9]. More recently, full genome sequencing has been used to genotype ILTV, but these studies are still very few [3, 15, 16, 19–23]. The genetic variation across the full genome sequences indicated that the field strains were either closely related to CEO vaccines and highly correlated each other, even though they derived from two distinct epidemics of ILT occurred in a period spanning almost 30 years and with different ILT control programs implemented by the Italian poultry industry. Therefore, we recommend that ILTV strain identification should be based on full genome analysis.

### Phylogenetic Analysis

**Fig 2:**
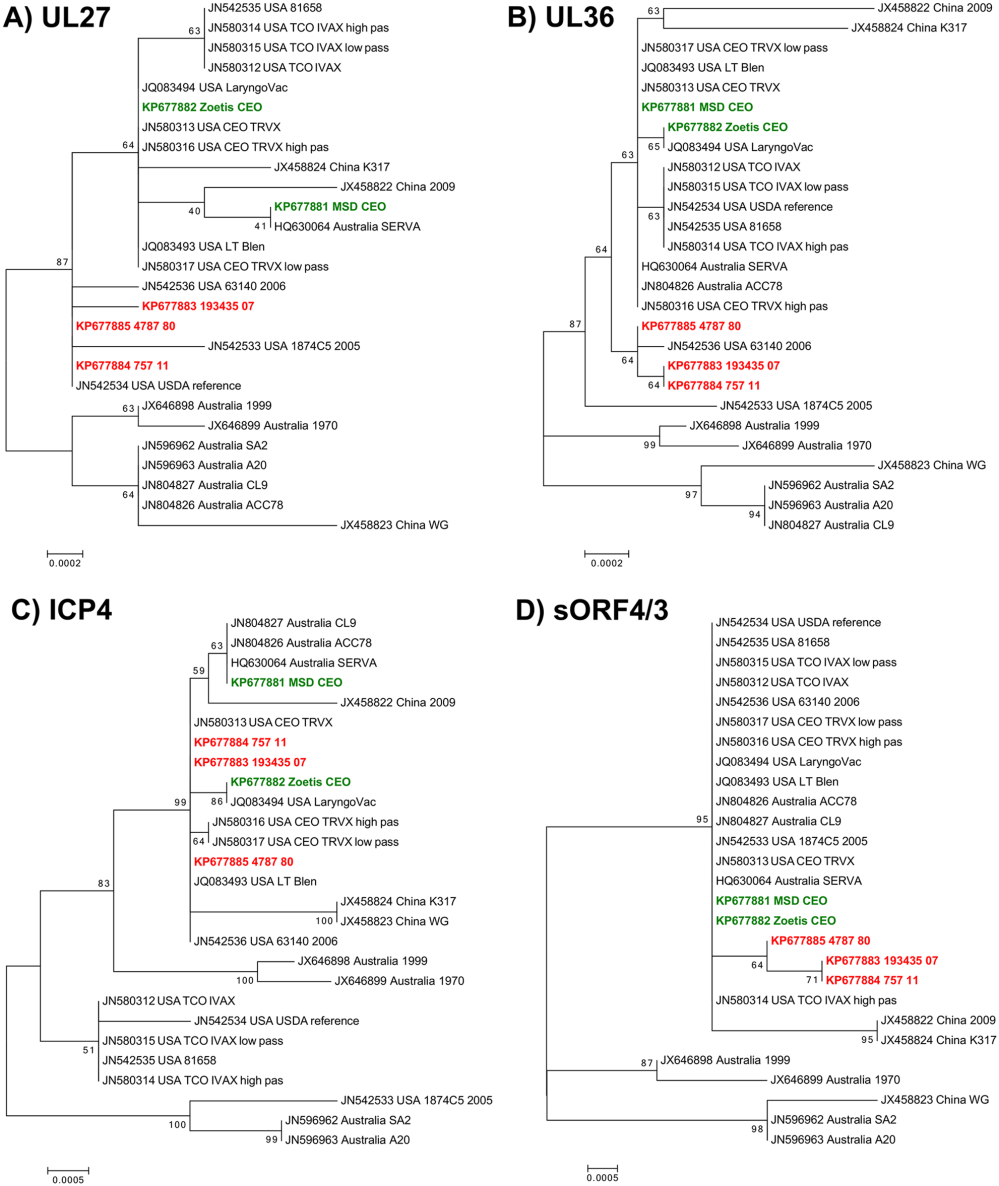
Evolutionary relationships of taxa. The evolutionary history was inferred using the Maximum Likelihood method based on the Tamura-Nei model [28] on four different genes: UL27 (A), UL36 (B), ICP4 (C), and sORF4/3 (D). ILTV genomes sequenced in this study are highlighted in red (field strains) and green (vaccine strains). The percentage of replicate trees in which the associated taxa clustered together in the bootstrap test (1000 replicates) are shown next to the branches [45]. All positions containing gaps and missing data were eliminated. Evolutionary analyses were conducted in MEGA6 [27].

Some of the genes with the highest variability among the ILTV complete genomes were selected to perform phylogenetic analyses. According to Lee et al. [20] and to our data, we chose the UL27, UL36, ICP4, and sORF4/3 genes: their nucleotide sequences, obtained from all the complete ILTV genomes available in GenBank, were analysed with the Maximum Likelihood method and the resulting trees are reported in Fig 2. In all cases, the Australian vaccine strains SA2 and A20 clustered separately from the US and European genomes, with the only exception represented by the virulent strain 1874C5 in the ICP4-related tree (Fig 2C). On the other hand, the US and European CEO vaccines were always contained in a common cluster, which included also other virulent ILTV strains from China (LJS09 and K317), Australia (ACC78), and USA (63140). Also García et al. [16] and Menendez et al. [6] recognized a distinct phylogenetic group including CEO vaccines and the US 63140 strain, suggesting that this strain was a revertant of CEO vaccines. As regards the genomes sequenced in this study, the Zoetis and MSD vaccines clustered always with the other CEO vaccines, with the MSD showing a closer relationship to the Serva strain, while the three field isolates 4787/80, 193435/07 and 757/11 grouped together (Fig 2B and 2D), even though separated by a quite long spanning time. Overall, this phylogenetic analysis suggests that the Italian field strains may be closely related to CEO vaccines, even if the 4787/80 strain was isolated before the introduction of ILT vaccination in Italy.

In the last decade, molecular epidemiological studies have provided evidence that ILTV strains identical or closely related to CEO (and to a lesser extent to TCO) vaccines in America, Asia, Australia, and Europe [7–18] and ILTV strains originated by natural recombination of CEO vaccines (*i*.*e*. Serva and SA2/A20 vaccine strains) in Australia [15, 20] have been the cause of ILT outbreaks, even in regions of the world where live attenuated vaccines were not used [9, 11, 12, 43].

**Fig 3:**
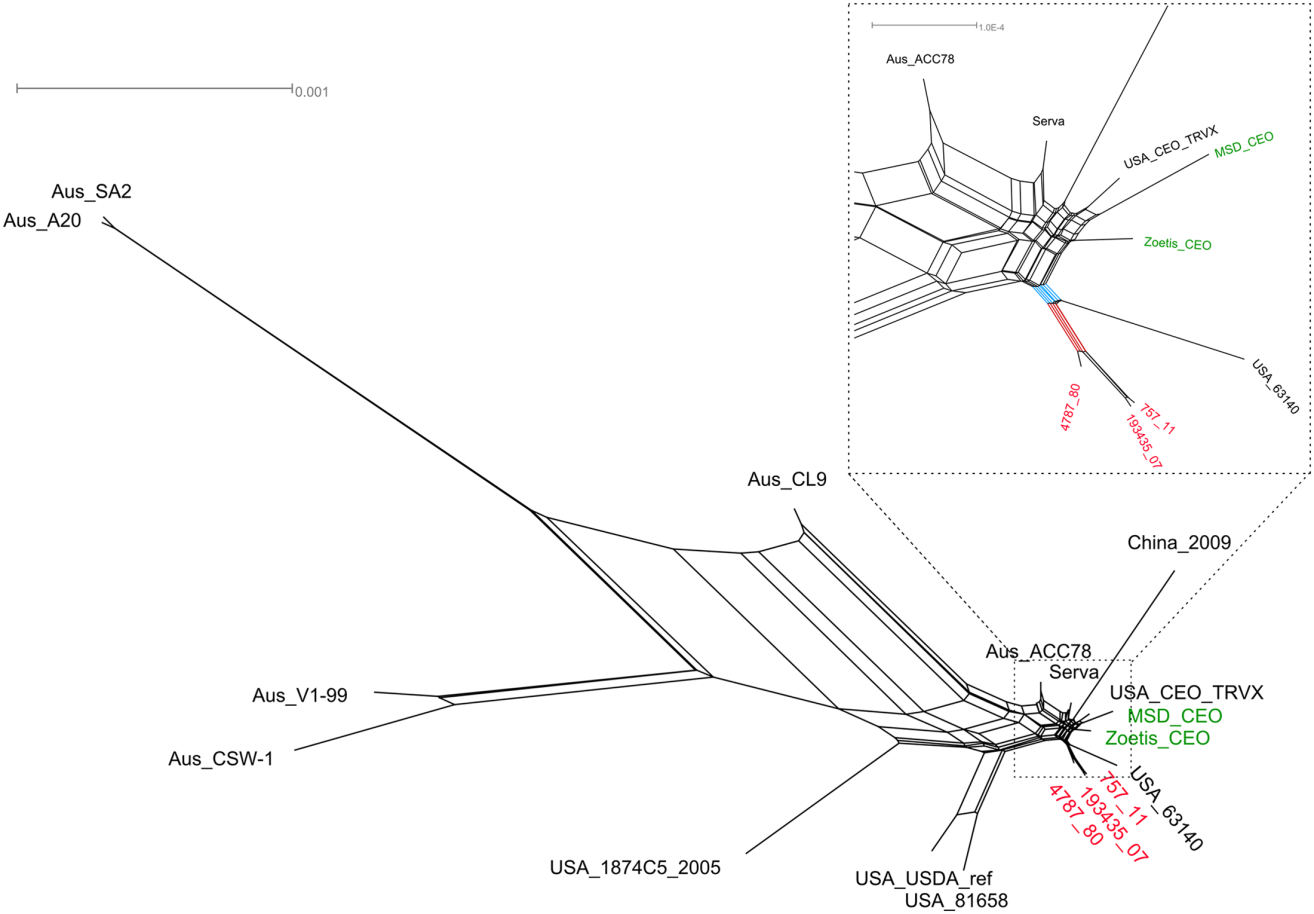
Split network showing reticulate evolutionary events of ILTV genomes. The network was obtained from the nucleotide sequences of Unique Long, Internal Repeat and Unique Short regions of ILTV genome using SplitsTree4 [46] based on the neighbor-net method [47]. The Italian field strains (in red) and the vaccine strains (in green) are closely related to the American CEO vaccines; in the zoomed inset, the split separating the field strains from all the other genomes is highlighted in red, while the one grouping them to the American virulent strain 63140 is in blue.

In order to obtain a global representation of the relationships existing among the currently known ILTV genomes, including the presence of recombinants, we generated a split network with 18 full genomes (Fig 3). The network confirms the close relationship between the Italian field strains and the CEO vaccines; nevertheless, the Italian isolates grouped together in a single cluster, and a further split included also the US 63140 field strain. This result, together with the high sequence similarity existing between the historical Italian strain and the CEO vaccines, suggests that an already existing CEO revertant ILTV strain could have been present in Italy at that time and be the cause of the epidemic of the ‘80s. A recent study of Lee et al. [20] demonstrated that the Australian CEO vaccine SA2 was very similar to ILTV strains isolated from backyard flocks in the US, suggesting that this strain shared a common ancestor originated in the American continent and subsequently introduced into Australia. Therefore, it is reasonable to suppose that the precursor of the Italian ILTV wild-type strain might have been a CEO revertant, perhaps introduced into Italy from the US.

## Conclusions

This is the first study reporting the full genomic sequences of Italian ILTV field and vaccine strains and providing valuable data about genomic variation of divergent ILTV strains from a new geographical region, such as Europe. Several mutations were identified across the genomes: some previously reported as associated with ILTV pathogenicity, some novel and exclusive of the Italian strains. The comparison of the genomes of wild-type strains isolated during epidemics occurred across a quite long spanning time suggested that Italian ILT outbreaks might have been related to a CEO revertant ILTV strain, maybe generated in the past. Further studies are required to better define the molecular determinants associated with ILTV virulence/attenuation and to individuate definitive markers associated with the virulence phenotypes, as well as to clarify the molecular epidemiology of ILT, recently challenged by the circulation of strains originated from live attenuated vaccines, and to identify the origin of strains circulating in poultry flocks.

## Supporting Information

S1 Table Primers designed for preliminary PCR of the five ILTV genomes. Nt = nucleotide; Primers were designed on the NCBI ILTV reference sequence (Gallid Herpesvirus 1, GenBank accession no. NC_006623).
(DOC)

S2 Table Primers designed for PCR and sequencing of the IR and for nested PCR and sequencing of the IR/TR variants of the five ILTV strains. (DOC)

## References

[1] García M, Spatz S, Guy JS. Infectious Laryngotracheitis In: Swayne DE, Glisson JR, McDougald LR, Nolan LK, Suarez DL, Nair V, editors. Diseases of Poultry. Ames, USA: Iowa State Press; 2013a pp. 161–179.

[2] Davison AJ. Herpesvirus systematics. Vet Microbiol. 2010; 143: 52–69. 10.1016/j.vetmic.2010.02.014 20346601 PMC2995426

[3] Lee SW, Markham PF, Markham JF, Petermann I, Noormohammadi AH, Browning GF, et al First complete genome sequence of infectious laryngotracheitis virus. BMC Genomics. 2011a; 12: 197. 21501528 10.1186/1471-2164-12-197 PMC3110152

[4] Coppo MJ, Noormohammadi AH, Browning GF, Devlin JM. Challenges and recent advancements in infectious laryngotracheitis virus vaccines. Avian Pathol. 2013; 42: 195–205. 10.1080/03079457.2013.800634 23718807

[5] Dufour-Zavala L. Epizootiology of infectious laryngotracheitis and presentation of an industry control program. Avian Dis. 2008; 52: 1–7. 18459288 10.1637/8018-051007-Review

[6] Menendez KR, García M, Spatz S, Tablante NL. Molecular epidemiology of infectious laryngotracheitis: a review. Avian Pat. 2014; 43: 108–117. 10.1080/03079457.2014.886004 24460399

[7] Creelan JL, Calvert VM, Graham DA, McCullough SJ. Rapid detection and characterization from field cases of infectious laryngotracheitis virus by real-time polymerase chain reaction and restriction fragment length polymorphism. Avian Pathol. 2006; 35: 173–179. 16595312 10.1080/03079450600598244

[8] Ojkic D, Swinton J, Vallieres M, Martin E, Shapiro J, Sanei B, et al Characterization of field isolates of infectious laryngotracheitis virus from Ontario. Avian Pathol. 2006; 35: 286–292. 16854641 10.1080/03079450600815481

[9] Neff C, Sudler C, Hoop RK. Characterization of western European field isolates and vaccine strains of avian infectious laryngotracheitis virus by restriction fragment length polymorphism and sequence analysis. Avian Dis. 2008; 52: 278–283. 18646457 10.1637/8168-110107-Reg.1

[10] Oldoni I, Rodriguez-Avila A, Riblet A, García M. Characterization of infectious laryngotracheitis virus (ILTV) isolates from commercial poultry by polymerase chain reaction and restriction fragment length polymorphism (PCR-RFLP). Avian Dis. 2008; 52: 59–63. 18459297 10.1637/8054-070607-Reg

[11] Chacón JL, Ferreira AJP. Differentiation of field isolates and vaccine strains of infectious laryngotracheitis virus by DNA sequencing. Vaccine. 2009; 27: 6731–6738. 10.1016/j.vaccine.2009.08.083 19747995

[12] Chacón JL, Mizuma MY, Ferreira AJP. Characterization by restriction fragment length polymorphism and sequence analysis of field and vaccine strains of infectious laryngotracheitis virus involved in severe outbreaks. Avian Pathol. 2010; 39: 425–433. 10.1080/03079457.2010.516386 21154050

[13] Blacker HP, Kirkpatrick NC, Rubite A, O’Rourke D, Noormohammadi AH. Epidemiology of recent outbreaks of infectious laryngotracheitis in poultry in Australia. Aust Vet J. 2011; 89: 89–94. 10.1111/j.1751-0813.2010.00665.x 21323656

[14] Sadeghi M, Bozorgemehrifard MH, Keyvanfar H, Momtaz H, Shooshtari A, Charkhkar S. Differentiation of field isolates and vaccine strains of infectious laryngotracheitis virus by DNA sequencing. Afr J Microbiol Res. 2011; 5: 4112–4117.

[15] Lee SW, Markham PF, Coppo MJC, Legione AR, Markham JF, Noormohammadi AH, et al Attenuated vaccines can recombine to form virulent field viruses. Science. 2012; 337: 188 10.1126/science.1217134 22798607

[16] García M, Volkening J, Riblet S, Spatz S. Genomic sequence analysis of the United States infectious laryngotracheitis vaccine strains chicken embryo origin (CEO) and tissue culture origin (TCO). Virology. 2013b; 440: 64–74. 23537957 10.1016/j.virol.2013.02.007

[17] Kim HR, Kang MS, Kim MJ, Lee HS, Kwon YK. Restriction fragment length polymorphism analysis of multiple genome regions of Korean isolates of infectious laryngotracheitis virus collected from chickens. Poult Sci. 2013; 92: 2053–2058. 10.3382/ps.2013-03134 23873552

[18] Shehata AA, Halami MY, Sultan HH, Abd El-Razik AG, Vahlenkamp TW. Chicken embryo origin-like strains are responsible for Infectious laryngotracheitis virus outbreaks in Egyptian cross-bred broiler chickens. Virus Genes. 2013; 46: 423–430. 10.1007/s11262-012-0870-2 23288626

[19] Lee SW, Devlin JM, Markham JF, Noormohammadi AH, Browning GF, Ficorilli NP, et al Comparative analysis of the complete genome sequences of two Australian origin live attenuated vaccines of infectious laryngotracheitis virus. Vaccine. 2011b; 29: 9583–9587. 22044743 10.1016/j.vaccine.2011.10.055

[20] Lee SW, Devlin JM, Markham JF, Noormohammadi AH, Browning GF, Ficorilli NP, et al Phylogenetic and molecular epidemiological studies reveal evidence of multiple past recombination events between infectious laryngotracheitis viruses. PLoS ONE. 2013; 8: e55121 10.1371/journal.pone.0055121 23383306 PMC3562231

[21] Spatz SJ, Volkening JD, Keeler CL, Kutish GF, Riblet SM, Boettger CM, et al Comparative full genome analysis of four infectious laryngotracheitis virus (Gallid herpesvirus-1) virulent isolates from the United States. Virus Genes. 2012; 44: 273–285. 10.1007/s11262-011-0696-3 22173980

[22] Chandra YG, Lee J, Kong BW. Genome sequence comparison of two United States live attenuated vaccines of infectious laryngotracheitis virus (ILTV). Virus Genes. 2012; 44: 470–474. 10.1007/s11262-012-0728-7 22382591

[23] Kong C, Zhao Y, Cui X, Zhang X, Cui H, Xue M, et al Complete genome sequence of the first Chinese virulent infectious laryngotracheitis virus. PLoS One. 2013; 8: e70154 10.1371/journal.pone.0070154 23922947 PMC3726392

[24] Mandelli G, Vantellino G. La laringotracheite infettiva aviare. Rassegna sintetica. Riv Zoot Vet. 1981; 8: 328–345.

[25] Moreno A, Piccirillo A, Mondin A, Morandini E, Gavazzi L, Cordioli P. Infectious Laryngotracheitis in Italy: clinical cases, diagnosis and characterization by polymerase chain reaction—restriction fragment length polymorphism (PCR-RFLP) and sequence analysis. Avian Dis. 2010; 54: 1172–1177. 21313836 10.1637/9398-051910-Reg.1

[26] Larkin MA, Blackshields G, Brown NP, Chenna R, McGettigan PA, McWilliam H, et al Clustal W and Clustal X version 2.0. Bioinformatics. 2007; 23: 2947–2948. 17846036 10.1093/bioinformatics/btm404

[27] Tamura K, Stecher G, Peterson D, Filipski A, Kumar S. MEGA6: Molecular Evolutionary Genetics Analysis version 6.0. Mol Biol Evol. 2013; 30: 2725–2729. 10.1093/molbev/mst197 24132122 PMC3840312

[28] Tamura K, Nei M. Estimation of the number of nucleotide substitutions in the control region of mitochondrial DNA in humans and chimpanzees. Mol Biol Evol. 1993; 10: 512–526. 8336541 10.1093/oxfordjournals.molbev.a040023

[29] Thiry E, Meurens F, Muylkens B, McVoy M, Gogev S, Thiry J, et al Recombination in alphaherpesviruses. Rev Med Virol. 2005; 15: 89–103. 15546129 10.1002/rmv.451

[30] Thureen DR, Keeler CL Jr. Psittacid herpesvirus 1 and infectious laryngotracheitis virus: Comparative genome sequence analysis of two avian alphaherpesviruses. J Virol. 2006; 80: 7863–7872. 16873243 10.1128/JVI.00134-06 PMC1563825

[31] Connolly SA, Jackson JO, Jardetzky TS, Longnecker R. Fusing structure and function: a structural view of the herpesvirus entry machinery. Nat Rev Microbiol. 2011; 9: 369–381. 10.1038/nrmicro2548 21478902 PMC3242325

[32] Eisenberg RJ, Atanasiu D, Cairns TM, Gallagher JR, Krummenacher C, Cohen GH. Herpes virus fusion and entry: a story with many characters. Viruses. 2012; 4: 800–832. 10.3390/v4050800 22754650 PMC3386629

[33] Pavlova S, Veits J, Mettenleiter TC, Fuchs W. Identification and functional analysis of membrane proteins gD, gE, gI, and pUS9 of Infectious laryngotracheitis virus. Avian Dis. 2013; 57: 416–426. 23901755 10.1637/10332-082612-Reg.1

[34] Zhou G, Galvan V, Campadelli-Fiume G, Roizman B. Glycoprotein D or J delivered in trans blocks apoptosis in SK-N-SH cells induced by a herpes simplex virus 1 mutant lacking intact genes expressing both glycoproteins. J Virol. 2000; 74: 11782–11791. 11090178 10.1128/jvi.74.24.11782-11791.2000 PMC112461

[35] Muylaert I, Tang KW, Elias P. Replication and recombination of herpes simplex virus DNA. J Biol Chem. 2011; 286: 15619–15624. 10.1074/jbc.R111.233981 21362621 PMC3091170

[36] Weller SK, Kuchta RD. The DNA helicase-primase complex as a target for herpes viral infection. Expert Opin Ther Targets. 2013; 17: 1119–1132. 10.1517/14728222.2013.827663 23930666 PMC4098783

[37] Sandri-Goldin RM. The many roles of the highly interactive HSV protein ICP27, a key regulator of infection. Future Microbiol. 2011; 6: 1261–1277. 10.2217/fmb.11.119 22082288

[38] Fan WH, Roberts AP, McElwee M, Bhella D, Rixon FJ, Lauder R. The large tegument protein pUL36 is essential for formation of the capsid vertex-specific component at the capsid-tegument interface of herpes simplex virus 1. J Virol. 2015; 89: 1502–1511. 10.1128/JVI.02887-14 25410861 PMC4300765

[39] Le Sage V, Jung M, Alter JD, Wills EG, Johnston SM, Kawaguchi Y. The herpes simplex virus 2 UL21 protein is essential for virus propagation. J Virol. 2013; 87: 5904–5915. 10.1128/JVI.03489-12 23487471 PMC3648149

[40] Veits J, Mettenleiter TC, Fuchs W. Five unique open reading frames of infectious laryngotracheitis virus are expressed during infection but are dispensable for virus replication in cell culture. J Gen Virol. 2003; 84: 1415–1425. 12771409 10.1099/vir.0.18926-0

[41] Kirkpatrick NC, Mahmoudian A, O'Rourke D, Noormohammadi AH. Differentiation of infectious laryngotracheitis virus isolates by restriction fragment length polymorphic analysis of polymerase chain reaction products amplified from multiple genes. Avian Dis. 2006; 50: 28–34. 16617977 10.1637/7414-072205R.1

[42] Oldoni I, García M. Characterization of infectious laryngotracheitis virus isolates from the US by polymerase chain reaction and restriction fragment length polymorphism of multiple genome regions. Avian Pathol. 2007; 36: 167–176. 17479379 10.1080/03079450701216654

[43] Chacón JL, Núñez LF, Vejarano MP, Parra SH, Astolfi-Ferreira CS, Piantino Ferreira AJ. Persistence and spreading of field and vaccine strains of infectious laryngotracheitis virus (ILTV) in vaccinated and unvaccinated geographic regions, in Brazil. Trop Anim Health Prod. 2015; 47: 1101–1108. 10.1007/s11250-015-0834-3 25904510

[44] Krzywinski M, Schein J, Birol I, Connors J, Gascoyne R, Horsman D, et al Circos: an information aesthetic for comparative genomics. Genome Res. 2009; 19: 1639–1645. 10.1101/gr.092759.109 19541911 PMC2752132

[45] Felsenstein J. Confidence limits on phylogenies: An approach using the bootstrap. Evol. 1985; 39: 783–791. 10.1111/j.1558-5646.1985.tb00420.x 28561359

[46] Huson DH, Bryant D. Application of phylogenetic networks in evolutionary studies. Mol Biol Evol. 2006; 23: 254–267. 16221896 10.1093/molbev/msj030

[47] Bryant D, Moulton V. Neighbor-net: an agglomerative method for the construction of phylogenetic networks. Mol Biol Evol. 2004; 21: 255–265. 14660700 10.1093/molbev/msh018

[48] Zhao Y, Kong C, Cui X, Cui H, Shi X, Zhang X, et al Detection of infectious laryngotracheitis virus by real-time PCR in naturally and experimentally infected chickens. PLoS One. 2013; 8: e67598 10.1371/journal.pone.0067598 23840745 PMC3695875

