# Supplementary material for: Full Genome Sequence-Based Comparative Study of Wild-Type and Vaccine Strains of Infectious Laryngotracheitis Virus from Italy
Source: PLoS One. 2016 Feb 18;11(2):e0149529. doi: 10.1371/journal.pone.0149529 (PMC4758665; doi:10.1371/journal.pone.0149529)
Supplement: S1 Table — Nt = nucleotide; Primers were designed on the NCBI ILTV reference sequence (Gallid Herpesvirus 1, GenBank accession no. NC_006623). (DOC) [file pone.0149529.s001.doc]

**S1 Table.** Primers designed for preliminary PCR of the five ILTV genomes.

| **Nt start-end positions** | **PCR product size (bp)** | **Primer name** | **Sequence** |
| --- | --- | --- | --- |
| 1,329-10,616 | 9,287 | 1F | CGTCCCCTACTTGCTGAGAG |
|  |  | 1R | CTTGGTTGATGAACGTGGTG |
| 9,667-19,186 | 9,519 | 2F | GAGGTGCAGATGAGCAAACA |
|  |  | 2R | TTGCATGGAGATGGAAATGA |
| 16,968-25,415 | 8,447 | 3F | AGTAGAAGGTGCAGGGAGCA |
|  |  | 3R | GGTCCACGGTAGAACAGCAT |
| 21,757-31,047 | 9,290 | 4F | CTAACTTCCAGCGGCTGTTC |
|  |  | 4R | GTCTCAGGCTTCCAAAGCAC |
| 26,040-35,107 | 9,067 | 5F | CGTGGGGCAATTACAGACTT |
|  |  | 5R | CGTTTTTACCGCCAAGATGT |
| 34,670-44,085 | 9,415 | 6F | CTGCGGGCTAGACTAAATGC |
|  |  | 6R | CGCGTGCTCAGAATAAACAA |
| 42,803-54,499 | 11,696 | 7F | AAGGCAGCTATTTCGGTATCCGGT |
|  |  | 7R | TGGTATGATTTCAGCTCGTCGCCT |
| 53,382-61,557 | 8,175 | 8F | GCTCTGCCTACTACCGATGC |
|  |  | 8R | ACACAAACCCATCAGCAACA |
| 60,792-69,216 | 8,424 | 9F | GCTTGTGGTATGTCCGGAGT |
|  |  | 9R | AAAGCCCTCAACGCTAGTCA |
| 67,688-77,274 | 9,586 | 10F | GTATGCGAACGTCTGGGTTT |
|  |  | 10R | AGCGTGCCTTCGAACTAAAA |
| 76,534-87,427 | 10893 | 11F | GGTACAATTCGTCCCTCCTG |
|  |  | 11R | GCTGCTGCCAGACAATCATA |
| 86,529-94,645 | 8,116 | 12F | CACCACGGATGTTTTGACTG |
|  |  | 12R | AGCTTTCTCCGCCCTTAGTC |
| 91,945-10,1932 | 9,987 | 13F | GCGCAAAGTGACATTCTTCA |
|  |  | 13R | CGGTCCTGTTCTCAAAGCTC |
| 100,279-108,735 | 8,456 | 14F | CAAATCAAACAGAGCGCAAA |
|  |  | 14R | CAGGGCAAAATACGACTGGT |
| 105,988-114,095 | 8,107 | 15F | CGTCGCATTGCGGAAATGAGTTGA |
|  |  | 15R | GCTGGCCGGCAGCATTTATTTACT |
| 112,859-122,530 | 9,671 | 16F | TCTTTGTAGAGTGTCGCGGAAGCA |
|  |  | 16R | AAATCGCCCTCGATCCCTTCCATT |
| 120,162-129,687 | 9525 | 17F | GCGTTCTGTATTTGCAGGGCACTT |
|  |  | 17R | AATTTACGGTTTCACCCGCACACG |
| 127,141-136,453 | 9,312 | 18F | GCAGAGGCGTTGAAAGTAGG |
|  |  | 18R | GCGTACAATGGTTCGGTCTT |
| 132,017-141,563 | 9,546 | 19F | CGACTTGCGTTTCCACCGTCAAAT |
|  |  | 19R | GCGTTCTGTATTTGCAGGGCACTT |
| 138,571-148,536 | 9,965 | 20F | GCCTCTCGATTGCAACAACGTGAA |
|  |  | 20R | ACTGGCGTTGCTGGTGGTACTACT |

Nt = nucleotide; Primers were designed on the NCBI ILTV reference sequence (Gallid Herpesvirus 1, GenBank accession no. NC_006623).
