# Supplementary material for: Full Genome Sequence-Based Comparative Study of Wild-Type and Vaccine Strains of Infectious Laryngotracheitis Virus from Italy
Source: PLoS One. 2016 Feb 18;11(2):e0149529. doi: 10.1371/journal.pone.0149529 (PMC4758665; doi:10.1371/journal.pone.0149529)
Supplement: S2 Table — (DOC) [file pone.0149529.s002.doc]

**S2 Table.** Primers designed for PCR and sequencing of the IR and for nested PCR and sequencing of the IR/TR variants of the five ILTV strains.

| **Genomic region** | **PCR product size (bp)** | **Primer name** | **Sequence** |
| --- | --- | --- | --- |
| Internal Repeat (IR) | 14,647 | IR_F | GCAGAGATCTTACGCCCCTC |
|  |  | IR_R | AACGGAGACAAGACAACGCT |
| Variant 118,760 | 283 | F | GCGGGTCATCGACCAAAGA |
|  |  | R | GGAGAATGTCCCGATGTCGC |
| Variant 124,869 | 297 | F | GTCCGAACTCAAGGACGGTT |
|  |  | R | CTCTGCATGGTACGGCTCTC |
| Variant 126,302 | 283 | F | TCTCTTATTTCCCACGCGGC |
|  |  | R | CGTCGCAGAGTTGCGATATTT |
| Variant 114,091-114,098 | 288 | F | TTTCCCGCCACGTAAAGCTA |
|  |  | R | GTCAGCATGGTCCAACGAGA |
| Variant 115,469 | 291 | F | TCATGCGTCTATGGCGTTGA |
|  |  | R | CGCTTCACGTGGAAGGACTA |
| Variant 117,408 | 293 | F | TGTTTCTGGAACCGCTGGAG |
|  |  | R | TACTCTACGGGAGTCCTGGC |
